# Low switched memory B cells are associated with no humoral response after SARS-CoV-2 vaccine boosters in kidney transplant recipients

**DOI:** 10.3389/fimmu.2023.1202630

**Published:** 2023-10-24

**Authors:** Mariana Seija, Joaquin García-Luna, Florencia Rammauro, Andreína Brugnini, Natalia Trías, Rossana Astesiano, José Santiago, Natalia Orihuela, Catherine Zulberti, Danilo Machado, Cecilia Recalde, Federico Yandián, Ana Guerisoli, Javier Noboa, Sergio Orihuela, Lilian Curi, Emma Bugstaller, Oscar Noboa, Marcelo Nin, Sergio Bianchi, Adriana Tiscornia, Daniela Lens

**Affiliations:** ^1^ Centro de Nefrología, Hospital de Clínicas, Facultad de Medicina, Universidad de la República, Montevideo, Uruguay; ^2^ Departamento de Fisiopatología, Hospital de Clínicas, Facultad de Medicina, Universidad de la República, Montevideo, Uruguay; ^3^ Laboratorio de Citometría de Flujo, Departamento Básico de Medicina, Facultad de Medicina, Universidad de la República, Montevideo, Uruguay; ^4^ Departamento de Inmunobiología, Facultad de Medicina, Universidad de la República, Montevideo, Uruguay; ^5^ Institut Pasteur de Montevideo, Montevideo, Uruguay; ^6^ Centro de Trasplante INU, Hospital Italiano, Montevideo, Uruguay; ^7^ Centro de Trasplante, Hospital Evangélico, Montevideo, Uruguay; ^8^ Instituto Nacional de Donación y Trasplante, Hospital de Clínicas, Facultad de Medicina, Universidad de la República y Ministerio de Salud Pública, Montevideo, Uruguay

**Keywords:** SARS-CoV-2, vaccine, boosters, kidney transplant, memory B cell

## Abstract

**Introduction:**

The humoral response after SARS-CoV-2 vaccination and boosters in kidney transplant recipients (KTRs) is heterogeneous and depends on immunosuppression status. There is no validated immune measurement associated with serological response in clinical practice. Multicolor flow cytometric immunophenotyping could be useful for measuring immune response. This study aimed to study B- and T-cell compartments through Standardized EuroFlow PID Orientation after SARS-CoV-2 vaccination and their association with IgG SARS-CoV-2 seropositivity status after two doses or boosters.

**Methods:**

We conducted a multicenter prospective study to evaluate humoral response after SARS-CoV-2 vaccination in KTRs. *Heterologous regimen:* two doses of inactivated SARS-CoV-2 and two boosters of BNT162b2 mRNA (n=75). *Homologous vaccination*: two doses of BNT162b2 mRNA and one BNT162b2 mRNA booster (n=13). Booster doses were administrated to KTRs without taking into account their IgG SARS-CoV-2 seropositivity status. Peripheral blood samples were collected 30 days after the second dose and after the last heterologous or homologous booster. A standardized EuroFlow PID Orientation Tube (PIDOT) and a supervised automated analysis were used for immune monitoring cellular subsets after boosters.

**Results:**

A total of 88 KTRs were included and divided into three groups according to the time of the first detected IgG SARS-CoV-2 seropositivity: non-responders (NRs, n=23), booster responders (BRs, n=41), and two-dose responders (2DRs, n=24). The NR group was more frequent on mycophenolate than the responder groups (NRs, 96%; BRs, 80%; 2DRs, 42%; p=0.000). Switched memory B cells in the 2DR group were higher than those in the BR and NR groups (medians of 30, 17, and 10 cells/ul, respectively; p=0.017). Additionally, the absolute count of central memory/terminal memory CD8 T cells was higher in the 2DR group than in the BR and NR groups. (166, 98, and 93 cells/ul, respectively; p=0.041). The rest of the T-cell populations studied did not show a statistical difference.

**Conclusion:**

switched memory B cells and memory CD8 T-cell populations in peripheral blood were associated with the magnitude of the humoral response after SARS-CoV-2 vaccination. Boosters increased IgG anti-SARS-CoV-2 levels, CM/TM CD8 T cells, and switched MBCs in patients with seropositivity after two doses. Interestingly, no seropositivity after boosters was associated with the use of mycophenolate and a lower number of switched MBCs and CM/TM CD8 T cells in peripheral blood.

## Introduction

Several studies have reported that kidney transplant recipients (KTRs) show a decreased antigen-specific humoral immune response after two doses of SARS-CoV-2 vaccines compared with the healthy population (1–12) Therefore, extra boosters were recommended to achieve seroconversion and produce high titers of the IgG anti-receptor-binding domain (RBD) of SARS-CoV-2 in KTRs (13–21).

We have previously demonstrated that a two-dose-two-booster-heterologous vaccination scheme combining inactivated virus vaccine (Sinovac®) and BNT162b2 mRNA (Pfizer/BioNTech) improved humoral response in KTRs. Additionally, we have demonstrated that the seroconversion achieved with this scheme was approximately 70%, similar to the response obtained with 2 doses-1-booster-homologous BNT162b2 mRNA vaccination (20, 21).

Several reports have shown that seroconversion depends on the immunosuppression treatment. KTRs who received mycophenolate were less likely to seroconvert, whereas KTRs on mTOR inhibitors such as everolimus had higher SARS-CoV-2 IgG levels (1–10, 20, 22). B cell-depleting therapy with rituximab (RTX) also reduced the humoral response (22–24). Other clinical features associated with diminished humoral response to vaccination were a recent kidney transplant, advanced age, and impaired kidney function (20–24).

Immunological response after SARS-CoV-2 vaccination is heterogeneous and there is no clinical tool to anticipate it in clinical practice. There is no specific post-transplant measure of immunosuppression burden. Clinicians rely on drug levels, viral screening, and patients’ features to predict the risk of rejection versus overimmunosuppression (25–27).

Multicolor flow cytometric (FC) immunophenotyping has become a key tool in the evaluation of immunological disorders and could be useful for measuring immune response in KTRs (28–30). We hypothesized that lymphocyte immune profiling could be associated with the humoral response after boosters.

B cell composition is altered after kidney transplantation and has been correlated with the SARS-CoV-2 vaccination response (26). Impaired B- and T-cell function in KTRs has been described with triple immunosuppression based on an antimetabolite (mycophenolate), a calcineurin inhibitor (cyclosporine or tacrolimus), and prednisone, and it is associated with the degree of immunosuppression (27).

Immunological memory in the humoral system is provided by memory B cells and bone marrow-resident long-lived plasma cells (PCs). Memory B cells (MBCs) play a critical role in antibody production. These cells are produced during the immune response by a germinal center (GC)-dependent or a GC-independent pathway. In the GC-independent pathway, naïve B cells become activated after encountering their antigen (e.g., vaccine) and undergo clonal expansion and affinity maturation to unswitched memory B cells and short-lived plasma cells (31, 32). Unswitched MBCs carry IgM and IgD isotypes on the surface. They migrate to GCs to undergo a class switch recombination to become switched MBCs or PCs (33). However, a small number of switched memory B cells can also be produced outside of germinal centers, in a GC-independent pathway (32, 33).

Vaccination also has the capacity to shape the memory CD8 T- cell pool (34). Memory CD8 T cells increase after vaccination and boosters and persist long-term (35). Memory T-cell repertoire can recirculate in tissues and potentially be more responsive after re-encountering the antigen (34). CD8+ T cells can continue to be protective when antibody titers decline (34). This study aimed to study B and T-cell compartments after SARS-CoV-2 vaccination and their association with the time of the first detected IgG SARS-CoV-2 seropositivity after two doses or boosters.

## Materials and methods

**Figure 1:**
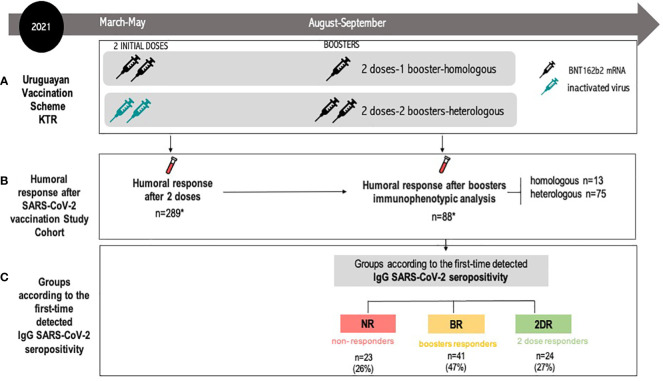
Flow diagram for the study participants. **(A)** Uruguayan Vaccination Scheme KTRs. heterologous regimen combination of two doses of inactivated SARS-CoV-2 (CoronaVac^®^) and two boosters of BNT162b2 mRNA. Homologous vaccination: two doses and one booster of BNT162b2 mRNA. **(B)** Humoral response after SARS-CoV-2 vaccination study cohort: the IgG anti-SARS-CoV-2 study was conducted 30 days after the second dose and after every booster. Immunophenotypic analysis was performed after the last booster. The study group was composed of 88 KTRs: *heterologous scheme* (n=75) and homologous scheme (n=13). **(C)** Groups according to the time of the first detected IgG SARS-CoV-2 seropositivity. Initial two-dose responders (2DRs): KTRs who were seropositive after two initial doses. Booster responders (BRs): KTRs who were seronegative after the initial two doses but did respond to the boosters (heterologous or homologous). Non-responders (NRs): those who did not respond after two initial doses and after boosters (heterologous or homologous). Seropositivity was defined as specific IgG antibodies against the receptor-binding domain (RBD) fragment of SARS-CoV-2 spike protein >10 BAU/ml. KTR, kidney transplant recipient.

We conducted a multicenter prospective study to evaluate humoral immune response after SARS-CoV-2 vaccination in KTRs after homologous and heterologous vaccination. Details of the Uruguay Vaccination Campaign are in the **Supplementary Material** (**Figure 1A**; **Supplementary Figure S1**).

The study group was composed of KTRs from all kidney transplant centers in Uruguay (INU-Hospital Italiano, Hospital Evangélico, and Hospital de Clínicas). The inclusion criteria were age >18 years old, kidney or kidney-pancreas transplant, informed consent, no prior PCR-confirmed COVID-19 during the vaccination and study period, and received two prior doses of CoronaVac^®^ or two doses of BNT162b2 as an initial vaccination and a subsequent homologous or heterologous booster according to the recommendations of the Uruguayan National Health Authority (**Figure 1A**).

The Uruguayan National Health Authority recommended vaccination for KTR patients with two initial doses of CoronaVac^®^ or two doses of BNT162b2 mRNA between March and June 2021. Later, in August 2021, booster doses with BNT162b2 mRNA were recommended for all solid organ transplant recipients without taking into account seroconversion status. As shown in **Figure 1A**, patients with two initial doses of BNT162b2 mRNA received one booster with the same vaccine (homologous vaccination) while patients receiving two doses of inactivated SARS-CoV-2 (CoronaVac^®^) received two boosters of BNT162b2 mRNA (heterologous vaccination). Therefore, all KTRs received either a homologous or heterologous scheme depending on the types of initial doses received (**Figure 1A**).

The humoral response was studied after the two initial doses and after every booster, as previously reported (20, 21) (**Figure 1B**). For the purpose of this study, we only included patients with available blood samples after two initial doses and after boosters with immunophenotypic analysis. Hence, the study group was composed of 88 KTRs who received either a heterologous scheme (n=75) or a homologous scheme (n=13). KTRs were divided into three groups according to the time of the first detected IgG SARS-CoV-2 seropositivity: non-responders (NR), booster responders (BR), and initial two-dose responders (2DR). Non-responders (NR) are those who did not respond after two initial doses and after boosters (heterologous or homologous). Booster responders (BR) are those who did not respond to the initial two doses but did respond to the boosters (heterologous or homologous). Finally, the initial two-dose responders (2DR) are KTRs who were seropositive after two initial doses (**Figure 1C**). A detailed flow chart of the study population is featured in **Figure 1**.

### Data collection and samples

Clinical data were recorded. The estimated glomerular filtration rate (eGFR) was estimated using the CKD-EPI formula.

Peripheral blood (PB) samples were collected 30–40 days after the last heterologous or homologous booster (**Supplementary Figure S1**). Freshly collected blood was centrifuged in the clot activator and gel tube (2,500 rpm, 15 min). Sera were separated and stored at −20°C until analysis. For immunophenotypic analysis, PB samples were collected in BD Vacutainer tubes containing K2EDTA (Becton/Dickinson, San Jose, CA) after the last heterologous or homologous booster. For each sample, a white blood cell (WBC) count was determined using a hematology analyzer (Unicel DxH 800 Beckman Coulter).

### IgG antibodies against the Receptor-Binding Domain

The level of serum-specific IgG antibodies against the Receptor-Binding Domain (RBD) fragment of SARS-CoV-2 Spike protein was determined using a COVID-19 IgG QUANT ELISA Kit (developed by Universidad de la República, Institut Pasteur de Montevideo, and ATGen) according to the manufacturer’s instructions. The assay has a sensitivity of 97.7% and a specificity of 96.2%. Quantitative test results were expressed in binding antibody units (BAU)/ml referred to by the First WHO International Standard for anti-SARS-CoV-2 immunoglobulin (NIBSC code: 20/136) used for assay calibration (20, 21, 36). Seropositivity was defined as specific IgG antibodies against the RBD fragment of SARS-CoV-2 Spike protein >10 BAU/ml. The value of 10 BAU/ml was defined as the assay cutoff, according to the manufacturer’s instructions. Samples above this value are considered positive (36).

### The standardized Euroflow PID orientation tube and supervised automated analysis

For flow cytometric analysis of major circulating leukocytes, we used the 8-color/12-marker standardized EuroFlow primary immunodeficiencies (PID) Orientation Tube (PIDOT) and a supervised automated analysis (Infinicyt, Cytognos, SL) (28–30). Briefly, non-nucleated red cells were lysed prior to staining, following the EuroFlow bulk lyse SOP (available at euroflow.org/protocols). Then, surface staining was performed with a reconstituted PIDOT lyophilized antibody cocktail (Cytognos) plus CD27 and CD45RA added as drop-in antibodies. Details about the specific antibody clones and fluorochrome-conjugated reagents used are provided in **Supplementary Table 1**. Staining and data acquisition of all samples were performed within 24 h after blood collection.

Data were acquired on a BD FACSCanto II™ flow cytometer (BD Biosciences), and instrument settings and data acquisition were performed according to the EuroFlow guidelines available at euroflow.org/protocols. Standard instrument settings were monitored by BD Cytometer Setup and Tracking (CS&T) beads (BD) and eight-peak Rainbow bead calibration particles (Spherotech, Lake Forest, IL). Data were exported as an FC standard file for further analysis.

Data Analysis: All FC standard data files were analyzed *automaticall*y using the Infinicyt module and the EuroFlow PIDOT database, with a special focus on the lymphoid populations. All major blood lymphocyte populations, including naïve B cells and post-GCs, memory B cells (MBCs), and plasma cells (PCs), as well as CD4 and CD8 maturation subsets, double negative (DN) gamma/delta T cells, and NK cells, were analyzed. Additionally, neutrophils, eosinophils, monocytes, CD16+ monocytes, and plasmacytoid dendritic cells were included.

The T-cell subsets were automatically analyzed and classified as naïve (CD27+CD45RA+), central memory/transitional memory (CM/TM; CD27+CD45RA-), effector memory (EM; CD27-CD45RA-) and terminally differentiated (TD; CD27-CD45RA+) CD4+ T cells; and naïve (CD27+CD45RA+), CM/TM (CD27+CD45RA-), EM (CD27-CD45-), and TD (CD27-CD45RA+) CD8+ T cells. Additionally, CD4/CD8 double negative and CD4/CD8 double-positive T cells were studied (35, 36). B-cell subsets were subdivided into naïve B cells (IgM+IgD+CD27−), unswitched memory B cells/plasma cells (unswitched MBC/PC; IgM+IgD+/−CD27+), and switched memory MBCs/PCs (IgM−IgD−CD27+) (28–30).

The absolute counts per microliter of the lymphocyte subsets identified by flow cytometry were calculated using a double platform approach with the absolute leukocyte counts determined before sample processing with a hematological analyzer population (Unicel DxH 800 Beckman Coulter) and the Infinicyt Statistics Configure tool.

All blood lymphocyte (sub)populations were compared with established Euroflow age-matched reference values. We classified all subpopulations below and above percentile 5 of the age-matched reference value because immunosuppression treatment decreases lymphocyte counts.

Reference populations were obtained from the Euroflow PIDOT database from multicentric healthy adult volunteers with no sign or suspicion of immunological or hematological diseases, including an abnormal infection rate or a known history of allergies (29, 30).

### Statistical analysis

Continuous variables were tested for normal distribution (Kolmogorov–Smirnov). Normally distributed variables were expressed as mean ± standard deviation (SD), non-normally distributed as median and interquartile range. Qualitative variables were expressed as numbers and percentages. The chi-square test was used to compare categorical variables. Continuous variables were compared using a t-test (normally distributed) or Kruskal–Wallis/Mann–Whitney test (non-normally distributed) with Bonferroni correction. As a normal distribution could not be demonstrated for any of the continuous variables, they were represented as median and interquartile range. Correlations of baseline levels of B cells, T cells, and anti-RBD IgG were calculated using Spearman’s rank correlation. *P*<0.05 was considered statistically significant. IBM^®^ SPSS^®^ version 22 (Chicago, IL) statistical software was used for statistical analyses Graph Pad 8 was used to construct charts.

## Results

Baseline patient characteristics according to the first time of detected IgG SARS-CoV-2 seropositivity are detailed in **Table 1**. Only 27% of KTR patients achieved seropositivity after the initial two doses of vaccination (2DR), whereas in most KTRs, seroconversion was observed after boosters (BR, 47%). Meanwhile, 26% of KTR patients remained seronegative (NR group).

**Table 1:** Clinical characteristics of patients according to the time of the first detected IgG anti-RBD SARS-CoV-2 seropositivity.

Variable	Time of the first detected IgG SARS-CoV-2 Seropositivity			p-value
	NRs ^Non-responders^	BRs ^Booster responders^	2DRs ^Two-dose responders^	
**N, (%)**	23(26%)	41(47%)	24 (27%)	
**Vaccine scheme** **Heterologous, n (%)** **Homologous, n (%)**	20 (87%) 3 (13%)	36 (87.8%) 5 (12.2%)	19 (79.2%) 5 (20.8%)	0.615
**Age years, median (IQR)**	55(47-55)	61 (43-54)	54 (39-53)	0.705
**Sex, men n (%)**	9 (39%)	13 (32%)	10 (41%)	0,687
**Comorbidities, n (%)** **Stroke** **Ischemic heart disease** **Peripheral arteriopathy**	1 (4%) 0 0	1 (2%) 3 (7%) 1 (2%)	0 0 0	0.604 0.169 0.560
**Diabetes mellitus, n (%)**	5 (22%)	12 (29%)	3 (13%)	0.295
**Time since transplantation: months,** **median (IQR)**	42 (31-110)	67 (31-158)	70 (27-42)	0.485
**Triple immunosuppression, n (%)**	20 (87%)	27 (66%)	13 (54%)	0.049
**Antimetabolite, n (%)** **None** **Mycophenolate** **Azathioprine**	1 (4%) 22 (96%) 0 (0%)	6 (15%) 33 (80%) 2 (5%)	9 (37%) 10 (42%) 5 (21%)	0,000
**Calcineurin inhibitors, n (%)** **None** **Tacrolimus** **Cyclosporine**	1 (4%) 19 (83%) 3 (13%)	4 (10%) 28 (68%) 9 (22%)	3 (12%) 20 (83%) 1 (4%)	0,306
**Prednisone, n (%)**	22 (96%)	97 (90%)	23 (96%)	0.593
**Everolimus, n (%)**	1(4%)	9 (22%)	8 (33%)	0,046
**Rituximab, n (%)**	0	2 (5%)	0	0.309
**Thymoglobulin, n (%)**	7 (30%)	5 (12%)	5 (21%)	0.203
**Rejection in the last 3 months, n (%)**	0	1 (2%)	0	0.561
**Lymphocyte count, cells/ul, median (IQR)**	1658 (951-2289)	1613 (1153-2243)	1618 (1474-2217)	0.416
**IgG RBD SARS-CoV-2 (BAU/mL) after 1 month homologous or heterologous boosters, median (IQR)**	2.5 (1-5)	291 (78-1047)	4229 (2773-12555)	0,000
**GFR ml/min/1.73m^2^, median (IQR)**	64 (55-47)	61(43-64)	62 (47-63)	0.637

Uruguayan Vaccination Scheme: heterologous regimen combination of two doses of inactivated SARS-CoV-2 (CoronaVac^®^) and two boosters of BNT162b2 mRNA. Homologous vaccination: two doses and one booster of BNT162b2 mRNA. The IgG anti-SARS-CoV-2 study was conducted 30 days after the second dose and after every booster. Immunophenotypic analysis was performed after the last booster. Groups according to the time of first detected IgG SARS-CoV-2 seropositivity: initial two-dose responders (2DR), KTRs with IgG anti-SARS-CoV-2 seropositivity after two initial doses; booster responders (BR), KTRs who were seronegative after the initial two doses but did respond to the boosters (heterologous or homologous); and non-responders (NR), those who did not respond after two initial doses and after boosters (heterologous or homologous). Seropositivity was defined as specific IgG antibodies against the receptor-binding domain (RBD) fragment of the SARS-CoV-2 spike protein >10 BAU/ml. KTR, kidney transplant recipient; eGFR, estimated glomerular filtration rate using the CKD EPI Formula; RBD, receptor-binding domain; SD, standard deviation; CNI, calcineurin inhibitors; IQR, interquartile range; triple immunosuppression, antimetabolite+calcineurin inhibitor+prednisone. Non-normally distributed as median and interquartile range (IQR).

The relative frequency of different maintenance immunosuppression regimens was different among the responder groups in our study. The proportion of patients on everolimus was higher in seroconversion groups than in non-responder patients (2DR, 33%; BR, 22%; and NR, 4%; p=0.046). However, patients with mycophenolate were the most frequent in the NR group (NR, 96%; BR, 80%; 2DR, 42%; p=0.000; **Table 1**). Age, diabetes, lymphocyte count, and glomerular filtration rate were similar in the three groups. The time from transplantation was lower in NR than in the BR and 2DR group, although this difference was not statistically significant (median 42, 67, and 70 months; p=0.485).

IgG RBD SARS-CoV-2 (BAU/mL) levels (shown in **Table 1**) were measured after 1 month of heterologous/homologous boosters. As expected, KTR patients in the 2DR group were the ones who had the highest levels of IgG anti-SARS-CoV-2 after the boosters (2DR 4,229 [2,773–12,555] vs. BR 291 (78–1,047) vs. NR 2.5 [1–5] BAU/ml) (**Table 1**). There were no differences in the IF analysis and in IgG anti-RBD titers between the homologous and heterologous KTRs (data not shown).

The composition and function of immune cell populations are age-dependent. Therefore, the analysis of the PIDOT tube includes age-specific reference ranges for the PIDOT database. In this study, we also analyzed the frequency of patients with values below the 5th percentile of the age-reference range for each subpopulation studied for the different response groups studied: NR, BR, and 2DR (29, 30). We divided lymphocyte subpopulations below and above the 5th percentile of the age-matched reference value because immunosuppression treatment decreases lymphocyte counts.

### B-cell populations according to the time of the first detected IgG SARS-CoV-2 seropositivity

**Figure 2:**
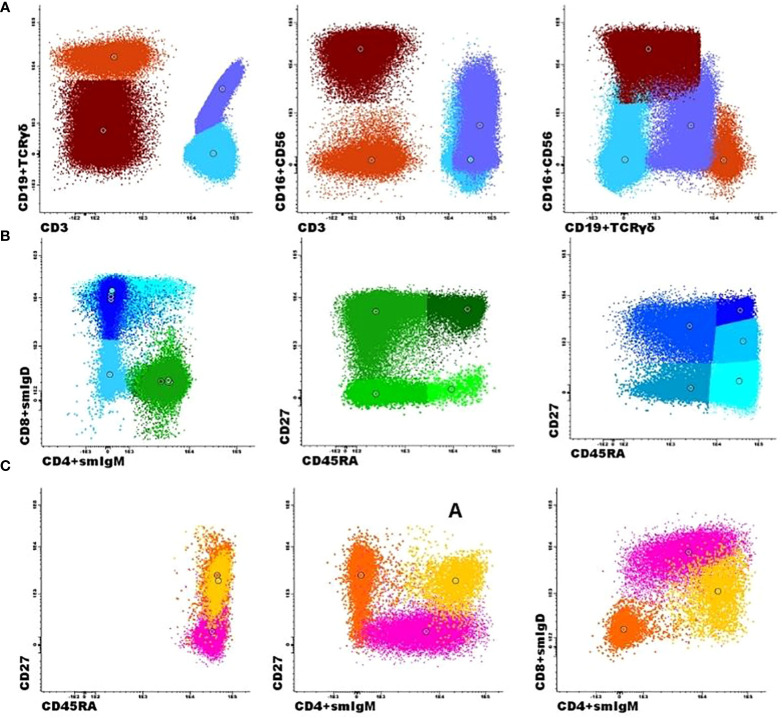
Flow cytometric analysis of B- and T-cell populations using the EuroFlow primary immunodeficiencies Orientation tube (PIDOT) and a supervised automated analysis (Infinicyt, Cytognos, SL). A representative clustering showing the supervised automated analysis of one patient. **(A)** After gating leukocytes as CD45+ and lymphocytes on FSC and SSC, the markers CD3 and CD19 in combination with TCRγδ and CD16 + 56 were used to define B cells (dark orange), TCRγδ+ T cells (lilac), TCRγδ- T cells (light blue), and NK cells (brown). **(B)** The T-cell subsets were further subdivided into naïve (CD27+CD45RA+; dark green), central memory/transitional memory (CM/TM; CD27+CD45RA-; bright green), effector memory (EM; CD27-CD45RA-; green), and terminally differentiated (TD; CD27-CD45RA+; light green) CD4+ T cells and into naïve (CD27+CD45RA+; dark blue), CM/TM (CD27+CD45RA-; blue), EM (CD27-CD45-; pale blue), TD (CD27-CD45RA+; turquoise), and dim positive CD27 effector (EffCD27dim; CD27int-CD45RA+; light blue) CD8+ T cells. **(C)** The B-cell subsets could be further subdivided into naïve (Naïve B cells, IgM+IgD+CD27−; pink), unswitched memory B cells/plasma cells (Unswitched MBCs/PCs; IgM^+^IgD^+/−^CD27^+^; yellow), and switched memory MBCs/PCs (IgM^−^IgD^−^CD27^+^; orange).

**Figure 3:**
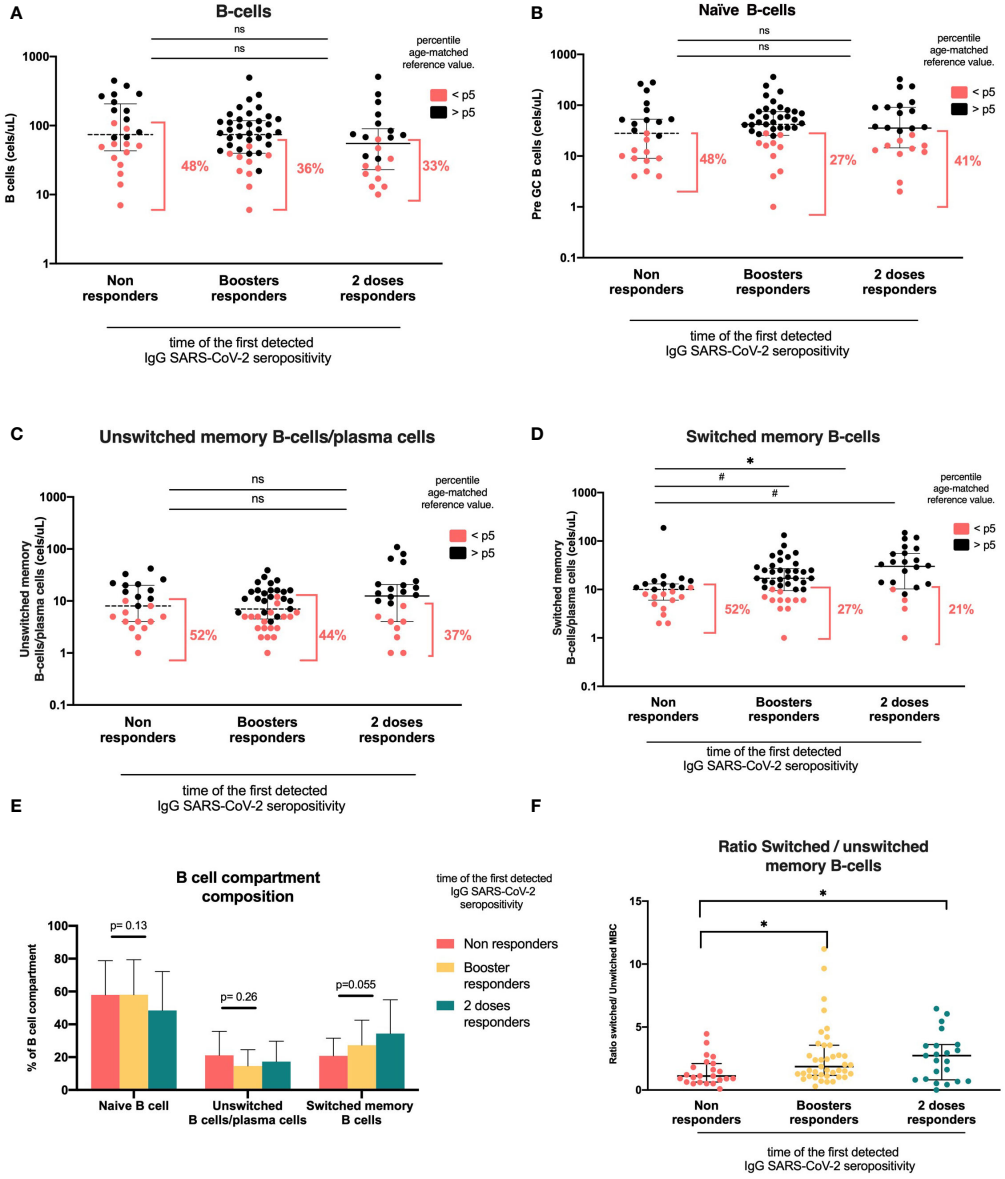
B-cell compartments according to the time of the first detected IgG anti-RBD SARS-CoV-2 seropositivity. Heterologous regimen combination of two doses of inactivated SARS-CoV-2 (CoronaVac^®^) and two boosters of BNT162b2 mRNA. Homologous vaccination: two doses and one booster of BNT162b2 mRNA. Groups according to the time of first detected IgG SARS-CoV-2 seropositivity: initial two-dose responders (2DRs), KTRs who were seropositive after two initial doses; booster responders (BRs), KTRs who were seronegative after the initial two doses but did respond to the boosters (heterologous or homologous); and non-responders (NRs), those who did not respond after two initial doses and after boosters (heterologous or homologous). **(A)**. B cells. **(B)**. Naïve B cells. **(C)** Unswitched memory B cells and plasma cells. **(D)** Switched memory B cells. **(E)** B-cell composition compartment. **(F)** Switched/unswitched memory B-cells ratio. In **(A–D)**, plots show the absolute number of B-cells using the PID orientation tube (PIDOT). Patients with B-cell counts below percentile 5 (p5) of the age-matched reference are in red and those above percentile 5 are in black. The horizontal bar in the graphs represents the median with the interquartile range. *p < 0.05: comparison of the proportions of lymphocytes below and above the 5^th^ percentile using the chi-square test. #p< 0.05: comparison of the absolute number of lymphocytes using Kruskal–Wallis with Bonferroni correction.

We analyzed all main circulating lymphocytes using the standardized PIDOT orientation tube and a supervised automated analysis that includes a database with age-matched reference values for each population studied (a representative clustering is shown in **Figure 2**). For B-cells, we analyzed three subsets: naïve B cells (IgM^+^IgD^+^CD27^−^), unswitched memory B cells or unswitched plasma cells (UnswMBCs/PCs; IgM^+^IgD^+/−^CD27^+^), and switched memory B cells or switched PCs (swMBCs/PCs; IgM^−^IgD^−^CD27^+^) (**Figure 3** and **Table 2**). Then, we analyzed the proportion of KTR patients falling below the 5^th^ percentile of the age-matched reference value for each population studied.

**Table 2:** Lymphocyte subpopulations according to the time of the first detected IgG anti-RBD SARS-CoV-2 seropositivity.

Variable	Time of the first detected IgG SARS-CoV-2 Seropositivity			p- value
	NRs ^Non-responders^	BRs ^Booster responders^	2DRs ^Two-dose responders^	
**B cells,** cell/ul, median (IQR)	63 (24-84)	77 (40-116)	74 (45-193)	0.214
**Naïve B cells,** cell/ul, median (IQR)	28 (9-53)	42 (26-74)	36 (15-90)	0.484
**Unswitched MBCs,** cell/ul, median (IQR)	8 (4-20)	7 (5-15)	12 (4-20)	0.590
**Switched MBCs,** cell/ul, median (IQR)	10 (6-13)	17 (10-25)	30 (10-55)	**0.005**
**T cells,** cell/ul, median (IQR)	1527 (687-1847)	1331 (863-1749)	1374 (1189-1934)	0.444
**CD4 T cells,** cell/ul, median (IQR)	631 (272-945)	539 (342-922)	714 (421-846)	0.568
**Naïve CD4 T cells,** cell/ul, median (IQR)	98 (38-255)	103 (39-190)	129 (58-242)	0.889
**CM/TM CD4 T cells,** cell/ul, median (IQR)	284 (143-395)	258 (163-418)	297 (208-442)	0.738
**Effector memory CD4 T cells,** cell/ul, median (IQR)	67(35-157)	82 (45-136)	88 (65-203)	0.356
**TD CD4 T cells,** cell/ul, median (IQR)	20 (6-60)	26 (7-77)	22 (5-42)	0.738
**CD8 T cells,** cell/ul, median (IQR)	605 (288-916)	546 (271-861)	568 (409-959)	0.471
**Naïve CD8 T cells,** cell/ul, median (IQR)	34(24-127)	37(22-97)	69(28-154)	0.505
**CM/TM CD8 T cells,** cell/ul, median (IQR)	98 (49-128)	93 (60-158)	166 (101-239)	**0.041**
**Effector memory CD8 T cells,** cell/ul, median (IQR)	35(13-80)	48(23-113)	56(21-117)	0.558
**TD CD8 T cells,** cell/ul, median (IQR)	41(17-75)	49 (19-108)	45(24-109)	0.843
**Double-negative T cells,** cell/ul, median (IQR)	6(3-11)	11(5-28)	10(3-29)	0.268
**NK cells,** cell/ul, median (IQR)	25 (12-51)	26(11-83)	38(21-69)	0.736
**Monocytes,** cell/ul, median (IQR)	609 (409-888)	605 (485-713)	539 (254-974)	0.667
**Plasmocytoid DC,** cell/ul, median (IQR)	7(5-10)	7(6-13)	8(4-17)	0.491

Non-normally distributed as median and interquartile range. GC, germinal center; MBCs, memory B cells; CM, central memory; TC, transitional memory; TD, terminally differentiated; DC, dendritic cell; NRs, non-responders; BRs, booster responders; 2DRs, two-dose responders.

We found that the only subset associated with seropositivity was swMBCs/PCs. Naïve B cell and unswMBC/PC absolute counts were similar among vaccination response groups. The proportion of KTR patients with unswMBC/plasma cells below the 5^th^ percentile was comparable in the three groups (naïve B cells, NR 48%, BR 27%, and 2DR 41%, p=0.201; unswMBC/PC, NR 52%, BR 44%, and 2DR 37%, p=0,597; **Figures 3B, C**, **Table 2**).

swMBC/PC counts in the NR group were lower than those in BR and 2DR (10 cells/ul (6–13), 17 cells/ul (10–25), and 30 cells/ul (10–55), respectively; p =0,017; **Table 2**). The proportion of patients with swMBC/PC cells below the 5^th^ percentile of the age reference was higher in the NR group than in the BR and 2DR groups (52%, 27%, and 21%, respectively; p=0.045; **Figure 3E**). Additionally, the ratio of swMBCs-PCs/unsw MBCs/PCs was lower in the NR group than in the responder groups (NR, 1.1; BR, 1.86; 2DR, 2.73; p=0.024; **Figure 3F**).

### T-cell compartment according to the time of the first detected IgG SARS-CoV-2 seropositivity

For T cells, we analyzed CD4 and CD8 maturation subsets naïve, central memory/transitional memory, effector memory, and terminally differentiated and DN gamma/delta T cells. T-cell values below the 5^th^ percentile of the age reference were more frequent in the NR and BR groups than in the 2DR group, although this difference was not statistically significant (NR, 26% and BR, 22% vs. 2DR, 8%; p=0.257).

**Figure 4:**
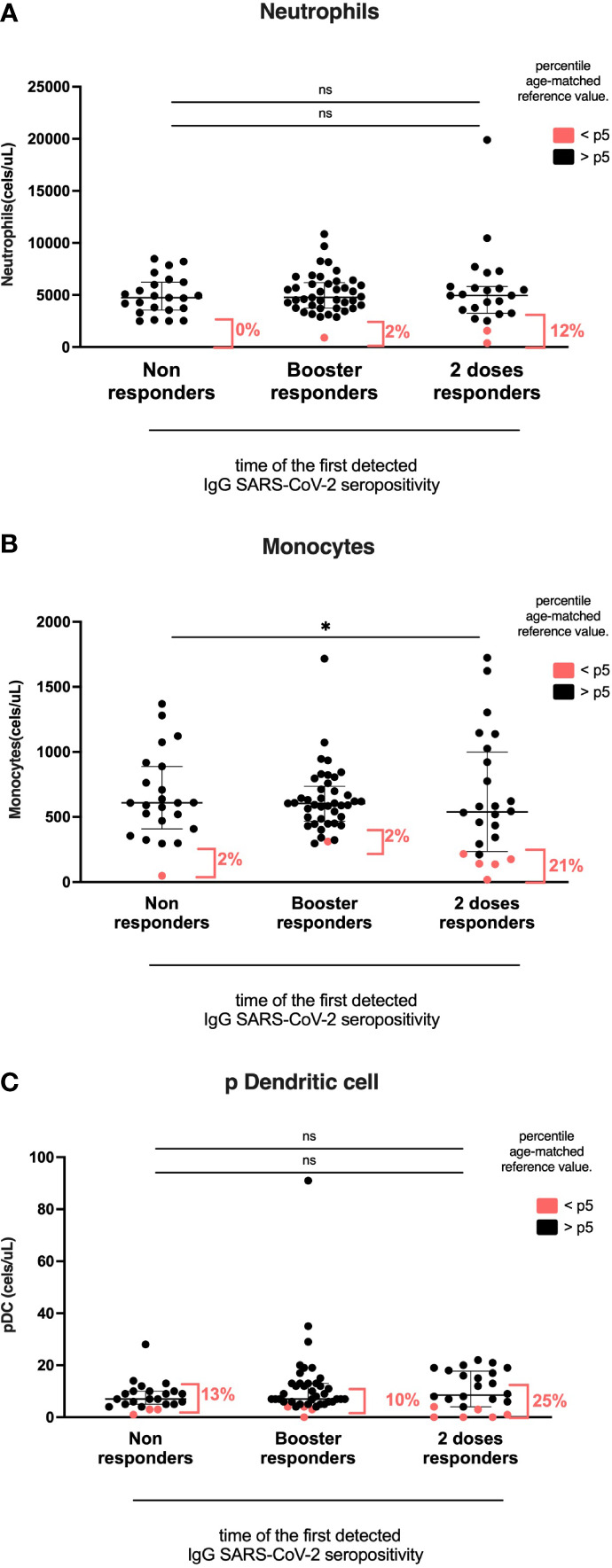
Myeloid cell compartment according to the time of the first detected IgG anti-RBD SARS-CoV-2 seropositivity. Heterologous regimen combination of two doses of inactivated SARS-CoV-2 (CoronaVac^®^) and two boosters of BNT162b2 mRNA. Homologous vaccination: two doses and one booster of BNT162b2 mRNA. Groups according to the time of the first detected IgG SARS-CoV-2 seropositivity: initial two-dose responders (2DRs), KTRs who were seropositive after two initial doses; Booster responders (BRs), KTRs who were seronegative after the initial two doses but did respond to the boosters (heterologous or homologous); and non-responders (NRs), those who did not respond after the initial two doses and after boosters (heterologous or homologous). **(A)** Neutrophils. **(B)** Monocytes. **(C)** Dendritic cells. In **(A–C)**, plots show the absolute number of cells using the PID orientation tube (PIDOT). Patients with cell counts below the fifth percentile of the age-matched reference are shown in red and those above the fifth percentile are shown in black. The horizontal bar in the graphs represents the median with the interquartile range. *p < 0.05, comparison of the proportions of cells below and above the 5^th^ percentile using the chi-square test. #p< 0.05, comparison of the absolute number of cells using Kruskal–Wallis with Bonferroni correction.

CD8 T-cells, naïve CD8 T-cells, effector memory, and terminally differentiated CD8 T-cell counts were similar among groups. However, the central memory/transitional memory (CM/TM) CD8 T-cell absolute count was significantly higher in the 2DR group than in the NR and BR groups (p=0.041; **Figure 4**, **Table 2**). CD4 T-cell and their subsets, double negative T-cell, and NK cell counts were similar among groups (**Table 2** and **Supplementary Figures 2B–E**).

### Myeloid cell compartment according to the time of the first detected IgG SARS-CoV-2 seropositivity groups

**Figure 5:**
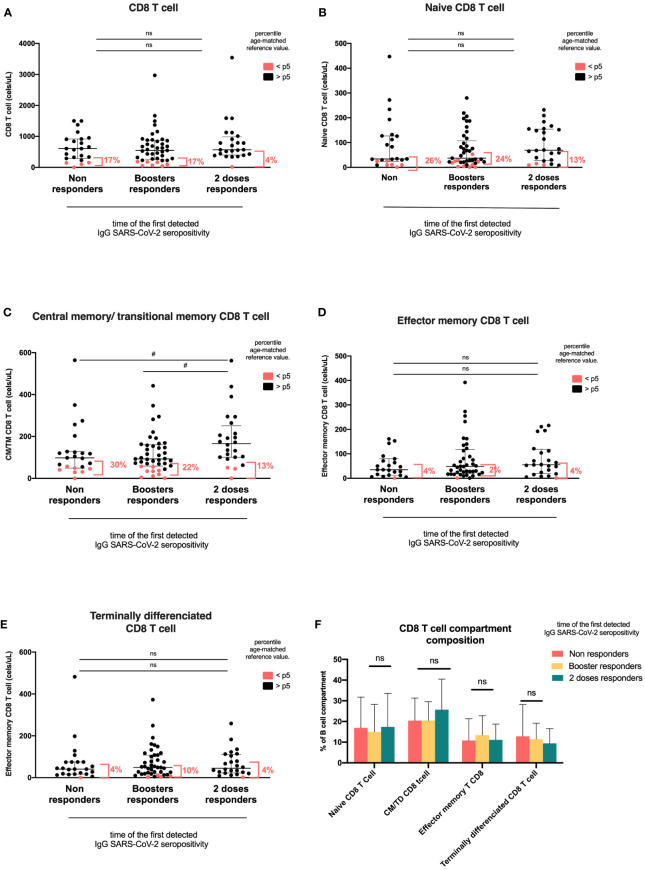
CD8 T-cell compartments according to the time of first detected IgG anti-RBD SARS-CoV-2 seropositivity. Heterologous regimen combination of two doses of inactivated SARS-CoV-2 (CoronaVac^®^) and two boosters of BNT162b2 mRNA. Homologous vaccination: two doses and one booster of BNT162b2 mRNA. Groups according to the time of the first detected IgG SARS-CoV-2 seropositivity: initial two-dose responders (2DRs), KTRs who were seropositive after two initial doses; booster responders (BRs), KTRs who were seronegative after the initial two doses but did respond to the boosters (heterologous or homologous); and non-responders (NRs), those who did not respond after two initial doses and after boosters (heterologous or homologous). **(A)** CD8 T cells. **(B)** Naïve CD8 T cells. **(C)** CM/TM CD8T cells. **(D)** Effector memory T CD8 cells. **(E)** Terminally differentiated CD8 T cells. **(F)** Composition of the CD8 T-cell compartment. In **(A–E)**, the plots show the absolute number of T cells using the PID orientation tube (PIDOT). Patients with a CD8 T-cell count below the fifth percentile of the age-matched reference are shown in red and those above the fifth percentile are shown in black. The horizontal bar in the graphs represents the median with the interquartile range. *p < 0.05, comparison of the proportions of lymphocytes below and above the 5^th^ percentile using the chi-square test. #p< 0.05, comparison of the absolute number of lymphocytes using Kruskal–Wallis with Bonferroni correction. CM, central memory; TM, transitional memory; TD, terminally differentiated.

The percentage of monocytes below the 5^th^ percentile of age reference was higher in the 2DR group than in the BR and NR groups (2DR, 21%; NR, 2%; BR, 2%; p=0.023). However, the absolute number of total monocytes and CD16^+^ monocytes was similar among groups (2DR, 539; no SC, 609; and BR, 605 cells/ul; p=0.277; **Figure 5**).

### Correlation between IgG SARS-CoV-2 levels, lymphocyte populations, and clinical parameters

**Figure 6:**
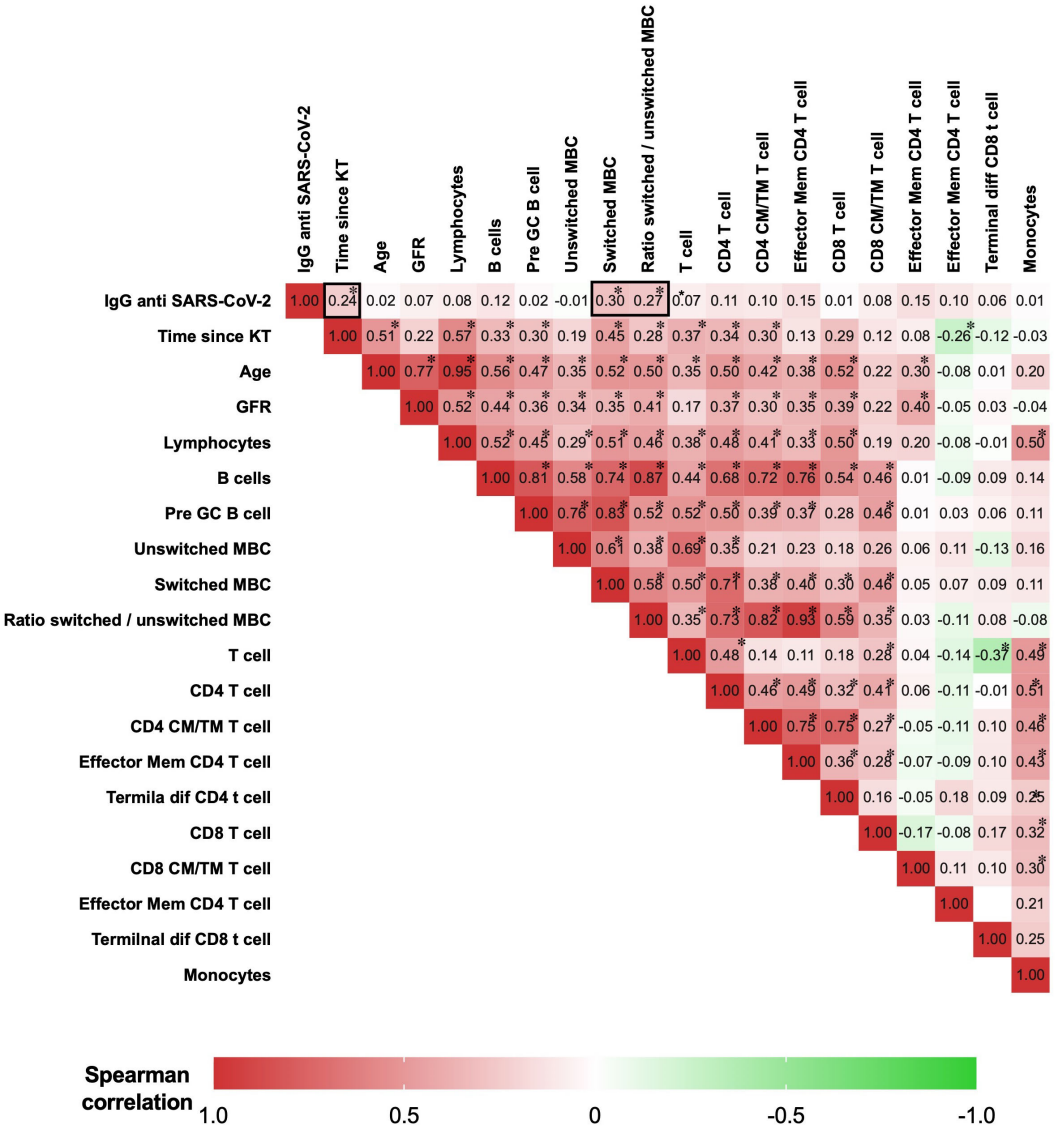
Matrix of Spearman’s correlation coefficients between IgG anti-SARS-CoV-2, lymphocytes, and clinical parameters. A color-coded correlation scale is provided. IgG anti-SARS-CoV-2 after a heterologous regimen combination of two doses of inactivated SARS-CoV-2 (CoronaVac^®^) and two boosters of BNT162b2 mRNA. Homologous vaccination, two doses and one booster of BNT162b2 mRNA. Correlations of continuous clinical parameters and absolute numbers of B cells, T cells, and anti-RBD IgG were calculated using Spearman’s rank correlation. *p<0.05 was considered statistically significant. KT, kidney transplant; GRF, glomerular filtration rate; GC, germinal center; CM, central memory; TM, transitional memory; TD, terminally differentiated; Mem, memory.

The Spearman correlation matrix was used to analyze the correlation between IgG anti-RBD SARS-CoV-2, lymphocyte absolute counts, and continuous clinical parameters. We summarized the representative correlations in **Figure 6**. There was a moderate correlation between IgG anti-RBD SARS-CoV-2 and switched memory B cells (Rho=0.3, p=0.005) and the ratio of switched/unswitched (Rho=0.27, p=0.011). Additionally, there was a weak correlation between the time of post-kidney transplantation and IgG anti-RBD SARS-CoV-2 (Rho=0.24, p=0.032). There was no association of IgG anti-RBD SARS-CoV-2 with age, glomerular filtration rate, or other T- and B-cell subpopulations.

## Discussion

An essential feature of vaccine evaluation and improvement is understanding the underlying immune mechanisms. There are several studies describing antibody responses to SARS-COV-2 vaccination and some of them also describe cellular responses (1–22, 37–57). However, there is little evidence concerning the response of T- and B-cell subpopulations in vaccinated KTRs. This study describes cellular immune responses following SARS-COV-2 vaccination and evaluates which features of this response correlate with the time of the first detected IgG SARS-CoV-2 seropositivity.

Immune profiles were studied after two doses-one booster-homologous and two doses-two boosters-heterologous SARS-CoV-2 vaccination. All patients received boosters independently of seropositivity status. Taking into account that the normal range of circulating lymphocyte subsets varies with age (28–30), we used the Euroflow PIDOT flow cytometry approach that includes a database with age-matched reference values. This approach enables us to compare lymphocyte subpopulation counts in KTR patients with the normal age-reference range. We calculated the frequency of patients with values below the 5^th^ percentile of the age-reference range for each subpopulation studied.

Here, we found that KTRs who respond to vaccines or boosters had a more prominent expansion of switched memory B cells after SARS-CoV-2 boosters. Non-responder KTRs had significantly lower switched MBC/PC cell counts than patients who responded. Additionally, the percentage of patients with swMBCs/PCs below the 5^th^ percentile of the normal range was 50% in the non-responder group compared with 20% in both seropositivity groups. This is in line with previous reports in which a reduced number of switched MBCs was associated with a low humoral response in KTRs and in autoimmune disease (22, 23, 26).

The presence of switched MBCs/PCs in the blood is an indicator of a functional germinal center reaction (35). In the germinal center, antigen-specific B cells undergo somatic hypermutation and affinity-based selection (58). SARS-CoV-2 mRNA-based vaccines induce a robust germinal center response in mice and humans (47, 58, 59). On the other hand, kidney transplant immunosuppression blunts T-B cooperation and decreases the humoral response (60–64).

KTRs in the 2DR group had higher switched MBC counts and IgG anti-SARS-CoV-2 levels after boosters. This may reflect a lower immunosuppression burden. We found that KTRs in this group were more frequently on mTOR inhibitors (everolimus) and less frequently on mycophenolate. This is in line with previous studies that have shown that humoral immune responses were better preserved with everolimus than with mycophenolate (58, 65, 66). Memory B cells seem better correlated with serological responses with mRNA vaccines and protein-based vaccines (67). The increase of antibodies after boosters is due to a significant expansion of memory B cells in the healthy population (58). Here, we showed that IgG anti-SARS-CoV-2 levels were associated with switched MBC counts, and higher levels were observed in the 2DR group than in the booster responders.

KTRs in the two-dose responder group also had more circulating CD8 CM/TM T cells than booster responders and non-responders. The central memory (CM) or effector memory (EM) differentiation states of CD4^+^ and CD8^+^ T cells have implications for durability and the responses upon antigen re-exposure. In immunocompetent individuals, low IgG levels after SARS-CoV-2 vaccination were associated with a low frequency of specific CD8+ T-cell memory and failed to control the delta and omicron variants of COVID-19 (34). This is in line with recent studies that showed IFNγ–producing CD8-T cells correlate with IgG titer and neutralization (68). In this study, anti-SARS-CoV-2 IgG levels were not correlated with CD8 CM/TM T cells.

In summary, we showed that boosters increase CM/TM CD8 T cells and switched MBCs in patients with a first detected IgG SARS-CoV-2 seropositivity after two doses. This could be explained by an increase in *SARS-CoV*-2 *specific* SARS-CoV-2 MBCs or CM/TM CD8 T cells; unfortunately, we did not measure these SARS-CoV-2 specific subpopulations.

In this study, seropositivity and IgG levels were not associated with different subpopulations of CD4 T cells. This was also observed in patients with other types of immunosuppression, such as hematological malignancies and solid organ transplants (69). By contrast, other researchers showed that low CD4 T-cell counts were associated with a diminished humoral response in patients with immunosuppression (19, 58, 70).

We also showed that two-dose responder KTRs had a higher percentage of monocytes below the 5^th^ percentile. Monocytes displayed a negative correlation with antibody titers in hemodialysis patients (71). In addition, some studies have demonstrated that inflammatory monocytes (CD14^+^, CD16^+^, and HLA-DR^+^) suppress vaccine responses (71–73). However, we did not find differences among groups in terms of CD16^+^ monocyte counts.

As we indicated previously, the present study did not assess SARS-CoV-2-specific B and T subsets. Nevertheless, the use of a single screening flow cytometry tube for immunodeficiency can enable a fast, standardized, and validated analysis of the immunophenotypic profile, making it a convenient method for studying the immune response in all clinical flow cytometry laboratories. The major advantage is that data can be fully exchanged between different clinical laboratories in any country. This information may be important to facilitate the development of a more effective vaccination scheme for patients on immunosuppression treatment. It should be noted that we used the PIDOT European database as a reference population, which may have differences with the Uruguayan population in lymphocyte counts due to environmental factors. However, it is worth noting that these discrepancies are expected to be minimal, given that Uruguayan individuals possess over 70% European genetic ancestry and have a similar diet and infection pattern to Europe (74).

In conclusion, switched memory B-cell counts in peripheral blood were associated with the humoral response after SARS-CoV-2 vaccination and boosters. Boosters increase IgG anti-SARS-CoV-2 levels, CM/TM CD8 T cells, and switched MBCs in patients with seropositivity after two doses. CD8+ T cells can continue to be protective when antibody titers decrease. Meanwhile, no seroconversion after boosters was associated with the use of mycophenolate and a lower number of switched MBCs and CM/TM CD8 T cells in peripheral blood. This information regarding B- and T-cell compartments could help the planning of a more effective vaccination scheme in KTRs based on the immune system response.

## Data availability statement

The datasets presented in this article are not readily available because it is sensitive data and not publicly available due to restrictions for containing information that could compromise the privacy of research participants. The data that support the findings of this study are available upon reasonable request keeping confidentiality. Requests to access the datasets should be directed to mariana.seija@gmail.com.

## Ethics statement

The studies involving humans were approved by Comitè de Etica del Hospital de Clìnicas (MSP 3535533). The studies were conducted in accordance with the local legislation and institutional requirements. The participants provided their written informed consent to participate in this study.

## Author contributions

MS and JN: acquisition, analysis, and interpretation of data, drafting the manuscript, and approval of the manuscript. JG, FR, AB, NT, and BS: acquisition and analysis of data. BS, JS, NO, CZ, DM, CR, RA, FY, AG, MN, SO, LC, EB, and ON: design of the study, enrolling patients, and reviewing the manuscript. AT and DL: interpretation of data, reviewing the manuscript, and approval of the manuscript. All authors contributed to the article and approved the submitted version.
