# Supplementary material for: Low switched memory B cells are associated with no humoral response after SARS-CoV-2 vaccine boosters in kidney transplant recipients
Source: Front Immunol. 2023 Oct 24;14:1202630. doi: 10.3389/fimmu.2023.1202630 (PMC10628322; doi:10.3389/fimmu.2023.1202630)
Supplement: Supplementary file 1 [file DataSheet_1.docx]

Supplementary Material

Low Switched Memory B Cells are associated with no humoral response after SARS-CoV-2 vaccine boosters in kidney transplant.

**Mariana Seija^1,2^**_†_**^,^ Joaquín García Luna^3^**_†_**, Florencia Rammauro^4,7^**_†_**, Andreína Brugnini^3^, Natalia Trías^3^, Rossana Astesiano^1^, José Santiago^1^, Natalia Orihuela^5^, Catherine Zulberti^5^, Danilo Machado^6^, Cecilia Recalde^6^, Federico Yandián^1^, Ana Guerisoli^1^, Javier Noboa^1,4^, Sergio Orihuela^5^, Lilian Curi^5^, Emma Bugstaller^6^, Oscar Noboa^1^, Marcelo Nin^1,5*^, Bianchi Sergio^2,3*^, Adriana Tiscornia^8^, Daniela Lens^3*^**

*** Correspondence:**Daniela Lens
daniela.lens@gmail.com

# Supplementary Data

**Supplementary Methods**

Uruguay vaccination campaign**:** In Uruguay, KTR were prioritized during the vaccination campaign. They receive 2 doses of inactivated SARS-CoV-2 (CoronaVac^®^) or 2 doses of BNT162b2 mRNA between march and April 2021. The initial doses depended on risk groups: healthcare workers and individuals over 70 years were vaccinated with 2 doses of BNT162b2 mRNA, meanwhile individuals between 18-70 years old received inactivated virus-based vaccine. In August 2021m the Uruguayan National Health Authority recommended booster doses with BNT162b2 mRNA vaccines for all solid organ transplantation recipients without taking into account seroconversion status. Therefore, KTR received a homologous or heterologous scheme. The *Heterologous vaccination* group received 2 doses of inactivated SARS-CoV-2 and 2 boosters of BNT162b2 mRNA 30 days apart each), the *Homologous vaccination grou*p received 3 doses of BNT162b2 mRNA

*Heterologous vaccination* group received 2 doses of inactivated SARS-CoV-2]. (CoronaVac^®^) and 2 boosters of BNT162b2 mRNA 30 days apart (n=92). *Homologous vaccination grou*p received 3 doses of BNT162b2 mRNA (n=17). Vaccine scheme depends on the type of two initial doses (Supplementary Figure S1).

**Data collection and measurements**

Blood samples were collected between 30 and 40 days after the second dose injection and 20 and 30 days of boosters. Freshly collected blood in the clot activator and gel tube was centrifuged (2500 rpm,15 min). Sera were separated and stored at -20°C until analysis.

Level of serum-specific IgG antibodies against the Receptor Binding Domain (RBD) fragment of SARS-CoV-2 Spike protein was determined using COVID-19 IgG QUANT ELISA Kit (developed by Universidad de la República, Institut Pasteur de Montevideo and ATGen Company), according to manufacturer’s instruction. The assay has a sensitivity of 97,7% and a specificity of 96,2%. Quantitative test results were expressed in Binding Antibody Units (BAU)/mL referred to the First WHO International Standard for anti-SARS-CoV-2 immunoglobulin (NIBSC code: 20/136) used for assay calibration. Seroconversion was defined as specific IgG antibodies against the Receptor Binding Domain (RBD) fragment of SARS-CoV-2 Spike protein > 10 BAU/mL.

The estimated glomerular filtration rate (eGFR) was calculated using the CKD-EPI formula.

The study was approved by the ethical institutional review board (MSP 3535533).

# Supplementary Figures and Tables

## Figure S1. Types of vaccination schemes and IgG anti-RBD measurement. Homologous vaccination: 3 doses of BNT162b2 mRNA vaccine 30 days apart; heterologous vaccination: 2 doses of inactivated SARS-CoV-2 vaccine (CoronaVac®) and two BNT162b2 mRNA boosters 30 days apart. Healthy control received 2 doses of inactivated SARS-CoV-2 vaccine (CoronaVac®) or 2 doses of BNT162b2 mRNA vaccine 28 days apart.

**Figure S2. CD4 T cell compartment according to IgG anti RBD SARS-CoV-2 status after the 3-dose homologous and 4-dose heterologous SARS-CoV-2 vaccination. a**). T cells. b) CD4 T cells c) Naïve CD4T cells d) CM/TM CD4 T cells e) Effector Memory CD4 T cells; f) Terminally differentiated CD4 T cell. Plots show the absolute number of T cells using the PID orientation tube (PIDOT); in red are shown patients with T cells count below percentile 5 of the age-matched reference and in black above percentile 5. * p < 0.05: comparison for proportions of lymphocytes below and above 5^th^ percentile using chi-square statistic; # p< 0,05 Comparison of absolute number of lymphocytes using Kruskal–Wallis with Bonferroni correction. CM: central memory, TM: transitional memory, TD: terminally differentiated.

**Supplementary table 1. Composition of the EuroFlow PID Screening tube**

| **Fluorochrome** | **BV421** | **BV510** | **FITC** | **PE** | **PerCP-Cy5.5** | **PC7-PE-Cy7** | **APC** | **APC-H7** |
| --- | --- | --- | --- | --- | --- | --- | --- | --- |
| **Marker** | CD27 | CD45RA | CD8  IgD | CD16  CD56 | CD4  IgM | CD19  TCRgd | CD3 | CD45 |
