# Supplementary figures and images for: Low switched memory B cells are associated with no humoral response after SARS-CoV-2 vaccine boosters in kidney transplant recipients
Source: Front Immunol. 2023 Oct 24;14:1202630. doi: 10.3389/fimmu.2023.1202630 (PMC10628322; doi:10.3389/fimmu.2023.1202630)

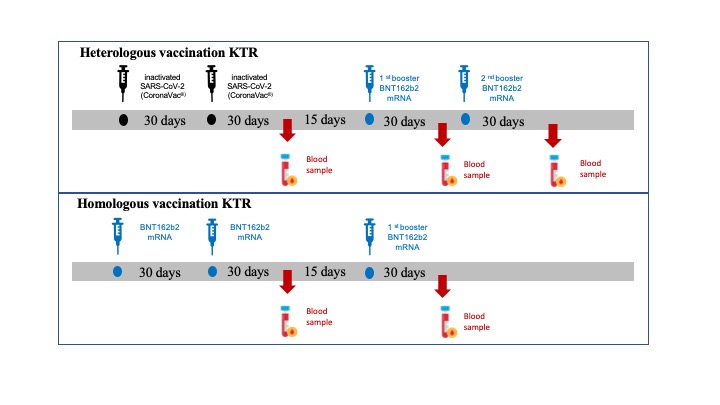

Supplement: Supplementary file 2 [file Image_1.jpeg]

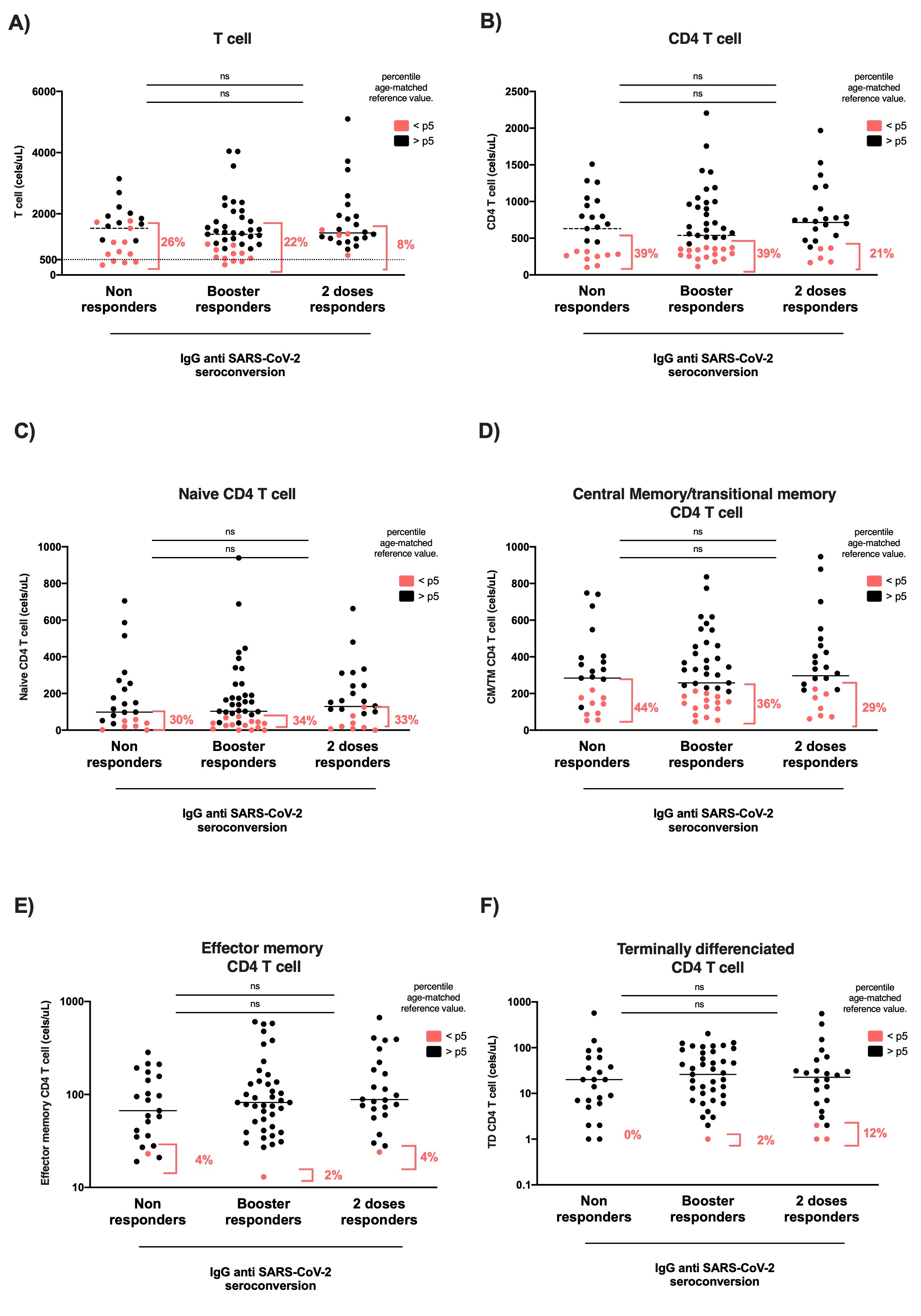

Supplement: Supplementary file 3 [file Image_2.jpeg]
